# Formation of Mono-Organismal and Mixed *Staphylococcus aureus* and *Streptococcus mutans* Biofilms in the Presence of NaCl

**DOI:** 10.3390/microorganisms13051118

**Published:** 2025-05-13

**Authors:** Yusuke Iwabuchi, Hiroko Yoshida, Shuichiro Kamei, Toshiki Uematsu, Masanori Saito, Hidenobu Senpuku

**Affiliations:** 1 Department of Pediatric Dentistry/Special Needs Dentistry, Division of Oral Health Sciences, Graduate School of Medical and Dental Sciences, Institute of Science Tokyo, Tokyo 113-8549, Japan; iwbcdpd@tmd.ac.jp; 2 Department of Orthodontics, Nihon University Dental School at Matsudo, Chiba 271-8587, Japan; mahi23014@g.nihon-u.ac.jp; 3 Department of Microbiology and Immunology, Nihon University Dental School at Matsudo, Chiba 271-8587, Japan; shu_dentistry@hotmail.com (S.K.); mato21013@g.nihon-u.ac.jp (T.U.); saito.masanori@nihon-u.ac.jp (M.S.)

**Keywords:** biofilm, membrane vesicle, *S. mutans*, NaCl, *S. aureus*

## Abstract

*Staphylococcus aureus*, an opportunistic bacterium found in the oral cavity, has been reported as a causative agent of infective endocarditis and pneumonia. Salt is an essential mineral for cell maintenance in the human body. This study was conducted to clarify how salt affects the formation of biofilms by *S. aureus* and *Streptococcus mutans*, pathogens implicated in dental caries. Bacteria were cultivated with various concentrations of NaCl on a 96-well microtiter plate in tryptic soy broth with 0.25% sucrose or 0.25% glucose (TSBs and TSBg, respectively) for 16 h. The effects of glucosyltransferase in *S. mutans* membrane vesicles (MVs) and extracellular DNA during biofilm formation were also analyzed. *S. aureus* biofilms were induced by 0.004–0.25 M NaCl but not by NaCl at concentrations greater than 0.25 M in TSBs. The mixed *S. aureus* and *S. mutans* biofilms gradually grew and were constructed by dead cells in a NaCl concentration-dependent manner in both TSBs and TSBg. Moreover, biofilms were slightly induced by glucan generation mediated by the glucosyltransferases in MVs under high-salinity conditions. The formation of mixed-species *S. aureus* and *S. mutans* biofilms increased in the presence of both extracellular DNA and MVs. Therefore, extracellular DNA, MVs, and dead cells are factors that promote *S. aureus* biofilm formation under harsh conditions containing NaCl. The sugar (sucrose and glucose) ingestion-induced *S. mutans* biofilm may be a risk factor for infection by opportunistic pathogens such as *S. aureus* in individuals who consume food and drinks containing high concentrations of salt.

## 1. Introduction

Oral biofilms on the tooth surface are biological communities of oral microorganisms that cause oral conditions such as dental caries and periodontal disease. Oral microorganisms metabolize the nutrients remaining in the oral cavity after eating, and biofilms are formed by their adhesion, proliferation, and aggregation on the tooth surface [1,2]. When a biofilm forms, the microorganisms within it are resistant to antibacterial substances, giving them an opportunity to survive in the oral cavity for a long time [3,4]. In recent years, the incidence of death due to aspiration pneumonia associated with oral biofilms has exceeded that of cerebrovascular diseases [5]. Oral and pharyngeal microorganisms are considered causative agents of pneumonia [6]. In developed countries, the proportion of elderly individuals is increasing due to declining birth rates and aging, and the number of elderly people that are bedridden is also increasing [7]. These bedridden elderly individuals have oral biofilms with more opportunistic pathogens, such as *Staphylococcus aureus* and *Candida albicans* [7,8]. *S. aureus*, which has also been detected in the oral cavity, has been reported as a causative agent of infective endocarditis and pneumonia in institutionalized elderly people [9,10]. The microorganisms that cause aspiration pneumonia are bacteria residing in the oral biofilm and oral mucosa [7,8]. Oral bacteria infect the lungs through saliva. In addition, *S. aureus*-infected immunocompromised hosts, such as those with diabetes and HIV, are at risk of developing systemic diseases. Notably, *S*. *aureus*, producing Panton–Valentine leukocidin (PVL), has been recognized as a causative agent of necrotizing pneumonia and severe musculoskeletal infections [11,12]. PVL is a potent cytolytic molecule with a unique ability to create pores in the cell membranes of human neutrophils and to induce the release of chemotactic factors [13]. In addition to pore-forming toxins, *S. aureus* expresses several superantigens that activate T cells by crosslinking the T-cell receptor with MHC class II molecules to disrupt the physiological immune response [14]. In co-occurring *S. aureus* lung infection and influenza, the proteases derived from *S*. *aureus* can cleave hemagglutinin, promote viral infection, and cause severe influenza [15].

Salt (NaCl and KCl, 0.9%) is used to flavor food and is an essential mineral necessary for the maintenance of cells in the human body. Certain ethnic groups in many countries effectively utilize salt in their diets. The concentration of salt is about 0.16% in human saliva from the oral cavity. However, salty foods (more than 3%) can also aggravate and increase dry mouth symptoms. When the mouth is dry, the normal commensal oral flora become a community that harbors many pathogens that cause oral diseases [16,17]. NaCl, at certain concentrations, exhibits bactericidal properties. Before toothpaste was used, NaCl was applied in the brushing of teeth. Additionally, salt is sometimes used in oral rinses and toothpastes to remove accumulated oral biofilms [18]. However, it has been reported that biofilm formation is promoted in media containing certain concentrations of NaCl [19,20]. This phenomenon is considered to be dependent on the salt concentration, but the detailed mechanism has not been elucidated. This phenomenon may be associated with the ability of *S. aureus* to survive under harsh conditions, including high salinity, as *S. aureus* is tolerant of high-salt concentrations, or with communication between *S. aureus* and the oral commensal bacteria in biofilms.

*Streptococcus mutans*, a pathogen of dental caries, forms biofilms composed of insoluble and soluble glucans induced by the principal enzymes glucosyltransferase B (GtfB) and glucosyltransferase C (GtfC) in the presence of sucrose [21]. Insoluble glucans synthesized by GtfB and GtfC are important virulence factors that strongly promote bacterial adhesion to tooth surfaces, their aggregation within the biofilm, and the development of an acidic environment [22,23]. Generally, Gram-negative and Gram-positive bacteria produce membrane vesicles (MVs) as they grow. Nucleotides, proteins, lipids, lipopolysaccharides, peptide glycans, lipoproteins, and enzymes, such as the toxic factor (toxins) GTFs, are associated with MVs [24,25]. MVs are produced abundantly by bacteria in nature and promote biofilm formation and interactions between bacteria and their growth environment [26]. Therefore, MVs play important roles in the communication between bacterial cells and are responsible for microbial interactions in host cells [25,26,27,28]. GtfC-containing MVs promote glucan-dependent biofilm formation by the initial oral colonizers on the tooth surface and are employed during cell-to-cell communication and the transition of bacteria from a non-virulent to a pathogenic state [23,29,30,31].

Extracellular DNA (eDNA) contributes to the development of biofilms and plays a number of important roles in bacterial adhesion to, and aggregation on, surfaces during the initial stage of biofilm formation [32,33]. Previous reports have shown that the absence of glucan and the presence of eDNA induce significant *S. mutans* biofilm formation on human saliva-coated hydroxyapatite disks upon raffinose supplementation [23,34]. eDNA may be required for *S. aureus* to initially attach to the surface, colonize it, and form biofilms with other bacterial species.

There are various factors, such as MVs with glucans and eDNA from dead cells from *S. mutans* [23,31], that may contribute to and affect the formation of *S. aureus* single- and mixed-species biofilms with *S. mutans* under high-salinity conditions. The purpose of this study was to clarify how these factors contribute to the formation of *S. aureus* single-species biofilms and *S. mutans* and *S. aureus* mixed-species biofilms and to identify the roles of these factors under harsh conditions, including those with high concentrations of NaCl. Studying how these factors affect biofilm formation under high-salinity conditions is important in the treatment of elderly immunocompromised patients with *S. aureus* infections.

## 2. Materials and Methods

### 2.1. Bacterial Strains and Culture Conditions

The bacterial strains *S. aureus* cowan I, *S. mutans* UA159 [29], and *S. mutans* UA159, as well as the *gtfBC* deletion mutant (*gtfBC*^−^) [30], were cultured and maintained in brain–heart infusion (BHI) broth supplemented with 0.086 M NaCl (Becton Dickinson and Company, Franklin Lakes, NJ, USA) at 37 °C in an atmosphere with 5% CO_2_ (gas pack: Mitsubishi Gas Chemical Co., Inc., Tokyo, Japan) for experimental use. The growth of *S. aureus* and *S. mutans* UA159 *gtfBC*^−^ was evaluated as the turbidity in BHI with and without various concentrations of NaCl by measuring the absorbance at 600 nm (OD_600_).

### 2.2. Collection of Human Saliva

Whole samples of saliva, stimulated by chewing paraffin gum, were collected over 5 min from 3 healthy human volunteers aged 25–31 years and pooled into ice-chilled sterile bottles. The samples were clarified via centrifugation at 10,000× *g* and 4 °C for 10 min, sterilized by filtering through 0.22 µm and 0.45 µm Millex-GP filter units (Merck kGaA, Darmstadt, Germany), and added to 96-well polystyrene microtiter plates (Sumitomo Bakelite, Tokyo, Japan) for biofilm formation assays. The use of human saliva to coat the plates in the study was approved by the Ethics Committee of Nihon University of Dental School (IRB approval number: EC21-029). Prior to enrolment, written informed consent was obtained from each subject according to the code of ethics of the World Medical Association (Declaration of Helsinki).

### 2.3. Extraction of MVs

*S. mutans* was cultivated in 1000 mL of BHI broth (pH 7.4) at 37 °C overnight in an atmosphere with 5% CO_2_. The initial cell density was 1.5 × 10^8^ in 1000 mL, and the final cell density was 1.5 × 10^11^ in 1000 mL. Additionally, separate BHI broth solutions (pH 6.0) were prepared with HCl, lactic acid, and acetic acid. Moreover, BHI broth solutions (pH 8.0) were prepared via the addition of NaOH and cultivated at 37 °C overnight in an atmosphere with 5% CO_2_. The MVs were prepared as described previously, with some modifications [21]. The culture supernatants were separated via centrifugation (6000× *g* for 20 min) and concentrated to >50 kDa via centrifugal filtration (Amicon Ultra 4, Merck kGaA, Darmstadt, Germany or VIVASPIN 20, Sartorius, Stonehouse, United Kingdom). Briefly, the concentrated MVs were filtered through polyvinylidene difluoride filter membranes (Merck kGaA) with pore sizes of 0.45 and 0.22 μm. The MV samples were centrifuged (150,000× *g* at 4 °C for 2 h) with a Beckman SW 41 Ti swinging bucket rotor and a Beckman optima L-90k ultracentrifuge (Beckman Coulter, South Kraemer Boulevard, Brea, CA, USA), and the pellets were suspended in sterile phosphate-buffered saline (PBS) (pH 7.2). The samples were also ultracentrifuged (150,000× *g* at 4 °C for 2 h), and the pellets were resuspended in 200 μL of sterile PBS for sample generation. The suspended samples were considered as MVs and used for the following experiments. The MV protein concentration was determined using a Bio-Rad protein assay kit (Bio-Rad Laboratories, Inc., Hercules, CA, USA).

### 2.4. Biofilm Formation Assay

Biofilms from each strain were developed in 96-well polystyrene microtiter plates (Sumitomo Bakelite) precoated with sterile human saliva at 4 °C overnight [23,34,35]. After washing with sterile PBS, the biofilm formation assays were performed according to a modified procedure [35]. Overnight cultures of *S. aureus* cowan I, *S. mutans* UA159, and *S. mutans* UA159.*gtfBC*^−^ in BHI broth were inoculated at a ratio of 1:100 in 200 µL of tryptic soy broth (TSB), supplemented with 0.086 M NaCl and either 0.25% sucrose (TSBs) or 0.25% glucose (TSBg), with or without 0.25 mg/mL MVs, in the presence of various concentrations of NaCl. MVs was confirmed via TEM and SEM in previous reports [23,31,36]. Three wells in each experiment were used for each concentration of NaCl, MVs, or mixture of salt and MVs. The concentration of MVs was confirmed to positively affect biofilm formation in a previous study [37]. Single cultures of *S. aureus* and *S. mutans* and mixed *S. aureus* and *S. mutans* UA159 or UA159.*gtfBC*^−^ bacterial cultures were tested in the presence of various concentrations of NaCl. TSBs and TSBg were used to observe glucan-dependent and glucan-independent biofilm formation, respectively. To determine how eDNA affects MV-dependent biofilm formation, 50 units/mL of DNase I (Roche Applied Science, Mannheim, Germany), which destroys DNA, was added to the biofilm formation assay components with 0.25 M NaCl. The plates were then incubated at 37 °C under aerobic conditions with 5% CO_2_ for 16 h. After incubation, the planktonic cells were removed by washing with distilled water (DW), and the adherent cells were stained with 0.25% safranin for 15 min to determine the extent of biofilm formation [35]. After washing twice with DW, the safranin was eluted from the biofilms with 70% (vol/vol) ethanol; then, the color intensity of the extracted safranin solution was used to determine the extent of biofilm formation. Biofilm formation was quantified by measuring the absorbance of the stained biofilms at 492 nm.

### 2.5. Observing Live Cells and Glucans During Biofilm Formation

The biofilms were stained with the FilmTracer *Live/Dead* Biofilm Viability Kit (Molecular Probes, Inc., Eugene, OR, USA), and SYTO 9 and propidium iodide were added to the biofilms, each at a final concentration of 5 µM. The glucans were labeled with an Alexa Fluor 647-dextran conjugate (Molecular Probes, Invitrogen Corp., Carlsbad, CA, USA) [22], which emits red fluorescence, whereas the nucleic acids in the bacterial cells were labeled with SYTO 9, which emits green fluorescence. A live/dead staining assay was performed in TSBg and TSBs, where red indicates dead cells, green indicates live cells, and yellow indicates a mix of live and dead cells.

The biofilms were incubated with the dyes at room temperature for 20–40 min before being imaged via confocal laser scanning microscopy (CLSM) (LSM700 Meta NLO, Carl Zeiss Inc., Jena, Germany). Two-dimensional (2D) images were acquired with a Plan-Apochromat 10×/0.45M27 objective lens. The confocal images of the biofilms were evaluated using ZEN (Carl Zeiss, ZEN2008) analysis software.

### 2.6. Statistical Analysis

The biofilm formation extents are expressed as the means ± standard deviations (SDs). In the biofilm assay, the significance of the differences between the bacteria with and without various concentrations of NaCl was determined via Quade non-parametric analysis of covariance (ANCOVA) and one-way analysis of variance (ANOVA) with Bonferroni correction (IBM SPSS statistics 28, IBM Corporation, Armonk, NY, USA). The biofilm comparisons between two conditions (NaCl vs. no NaCl, MV vs. no MV, and DNase I vs. no DNase I) were performed using Student’s *t*-test. A *p*-value of less than 0.05 was considered to indicate statistical significance. All experiments were repeated independently three times.

## 3. Results

### 3.1. Effects of NaCl Concentration on S. aureus and S. mutans Growth and Biofilm Formation

**Figure 1:**
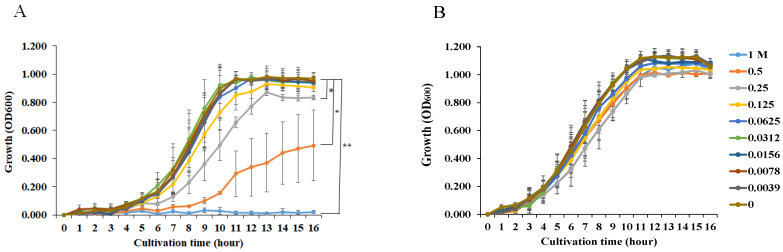
Effects of NaCl on the growth of *S. mutans* UA159.*gtfBC*^−^ and *S. aureus. S. mutans* UA159.*gtfBC*^−^ (**A**) and *S. aureus* cowan I (**B**) were cultivated at 0, 1/2^8^, 1/2^7^, 1/2^6^, 1/2^5^, 1/2^4^, 1/2^3^, 1/2^2^, 1/2^1^, and 1/2^0^ M NaCl dilutions in BHI. The OD_600_ of the bacterial cell suspension was measured after growth. The data are presented as the means ± SDs of three independent experiments. An asterisk indicates that there is a significant difference between the two groups in ANCOVA (*: *p* < 0.05, **: *p* < 0.01; various concentrations of NaCl vs. no NaCl).

**Figure 2:**
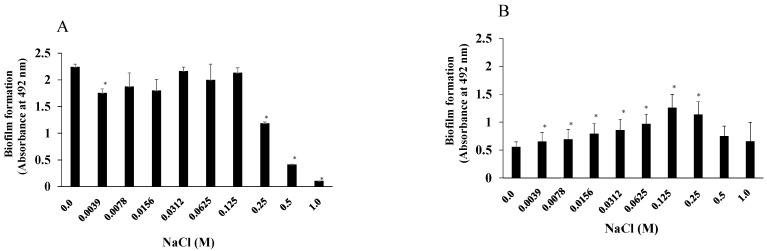
Effects of NaCl on the formation of single-species biofilms. *S. mutans* UA159 (**A**) and *S. aureus* cowan I (**B**) were cultivated at 0, 1/2^8^, 1/2^7^, 1/2^6^, 1/2^5^, 1/2^4^, 1/2^3^, 1/2^2^, 1/2^1^, and 1/2^0^ M NaCl dilutions in TSBs to assess biofilm formation. The data are presented as the means ± SDs of three independent experiments at 16 h after culture. An asterisk indicates that there is a significant difference between the two conditions in ANOVA with Bonferroni correction (*: *p* < 0.05; various concentrations of NaCl vs. no NaCl).

We examined the effects of NaCl on the growth of *S. mutans* UA159.*gtfBC*^−^ and *S. aureus* and found that more than 0.25 M NaCl significantly inhibited *S. mutans* UA159.*gtfBC*^−^ growth (Figure 1A). In contrast, the growth of *S. aureus* was not inhibited by any concentration of NaCl. To observe how NaCl affects the formation of a single-species biofilm, various concentrations of NaCl were applied in the biofilm formation assay using *S. mutans* and *S. aureus*. More than 0.25 M NaCl significantly inhibited the formation of *S. mutans* biofilms in TSBs (Figure 2A), and this was dependent on the inhibition of cell growth (Figure 1A). In contrast, 0.0039–0.25 M NaCl significantly induced *S. aureus* biofilm formation compared with that in the absence of NaCl (Figure 2B), and biofilm formation peaked at 0.125 M NaCl (Figure 2B).

### 3.2. Effects of NaCl on the Formation of Mixed-Species Biofilms

**Figure 3:**
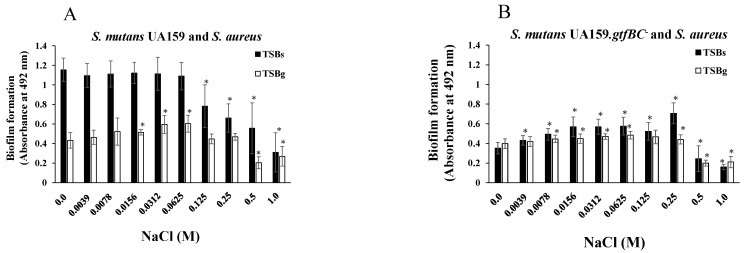
Effects of NaCl on the formation of mixed-species biofilms. *S. aureus* was inoculated with *S. mutans* UA159 (**A**) or *S. mutans* UA159.*gtfBC*^−^ (**B**) in TSBs or TSBg with various concentrations of NaCl to assess biofilm formation. The data are presented as the means ± SDs of three independent experiments at 16 h after culture starts. An asterisk indicates that there is a significant difference between the two conditions in TSBs or TSBg in ANOVA with the Bonferroni correction (*: *p* < 0.05, various concentrations of NaCl vs. no NaCl).

Next, mixtures of *S. aureus* and *S. mutans* were used in the biofilm formation assay in the presence of NaCl. TSBs and TSBg were used separately for cultivation to determine the effects of NaCl on *S. aureus* and *S. mutans* mixed-species biofilms in the presence and absence of glucan. *S. aureus* was mixed with *S. mutans* or *S. mutans* UA159.*gtfBC*^−^, lacking the insoluble glucan-synthesizing enzymes GtfB and GtfC, and added to 96-well microtiter plates precoated with human saliva with TSBs or TSBg containing various concentrations of NaCl. More than 0.125 M NaCl significantly inhibited the formation of the *S. mutans* and *S. aureus* mixed biofilms in TSBs (Figure 3A). In contrast, biofilm formation was significantly better in the presence of 0.0156 M, 0.0312 M, and 0.0625 M NaCl than in the absence of NaCl in TSBg (Figure 3A). More than 0.5 M NaCl significantly decreased the formation of mixed biofilms in TSBg. To completely eliminate water-insoluble glucan synthesis, *S. mutans* UA159.*gtfBC*^−^ was used instead of *S. mutans* UA159. The *S. aureus* and *S. mutans* UA159.*gtfBC*^−^ mixed biofilm gradually formed in a NaCl concentration-dependent manner, peaking at 0.25 M NaCl in TSBs (Figure 3B). However, biofilm formation was significantly inhibited with more than 0.5 M NaCl in comparison to 0 M NaCl. These results indicate that the formation of *S. aureus* and *S. mutans* UA159.*gtfBC*^−^ mixed biofilms in TSBs and TSBg was similar to that of *S. aureus* and *S. mutans* UA159 in TSBg. Therefore, in the absence of glucan, 0.125 M and 0.25 M NaCl significantly induced the formation of single-species *S. aureus* and mixed-species *S. aureus* and *S. mutans* UA159.*gtfBC*^−^ biofilms (Figure 2B and Figure 3B).

**Figure 4:**
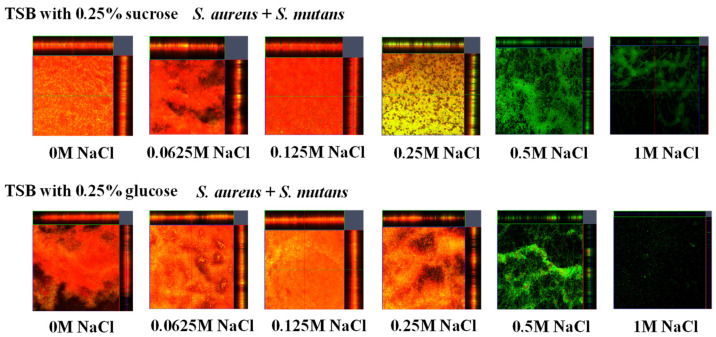
Effects of NaCl on the formation of mixed-species biofilms. *S. aureus* was inoculated with *S. mutans* UA159 in TSBs and TSBg with various concentrations of NaCl to assess biofilm formation. The biofilms were treated via live/dead staining and observed via confocal microscopy. Representative data from more than three independent experiments are presented in the images.

To observe the living and dead cells in the glucan-independent *S. aureus* and *S. mutans* mixed-species biofilms, a live/dead staining assay was performed in TSBg and TSBs. Dead cells were observed in the mixed-species biofilms in the presence of 0–0.25 M NaCl (Figure 4). Both living and dead cells were observed in the mixed-species biofilms with NaCl in the range of 0.0156–0.25 M. However, mainly live cells were observed with 0.5 M NaCl, but the amount of biofilm formation was small. Neither live nor dead cells were observed with 1 M NaCl. These results indicate that 0.0156–0.25 M NaCl, which significantly increased biofilm formation (Figure 3), induced dead and live cell-dependent mixed-species biofilms in the absence of glucan (Figure 4). Excess concentrations (>0.5 M) of NaCl inhibited the growth of *S. mutans* (Figure 1A) and did not induce dead cell-dependent biofilm formation in mixtures of *S. aureus* and *S. mutans.* Dead cells from *S. mutans* may support the biofilm formation of *S. aureus* in the range of 0–0.125 M NaCl in TSBs and 0–0.25 M NaCl in TSBg. A mix of live and dead cells was mainly observed at 0.25 M NaCl in TSBs.

### 3.3. Effects of MVs on Biofilm Formation

**Figure 5:**
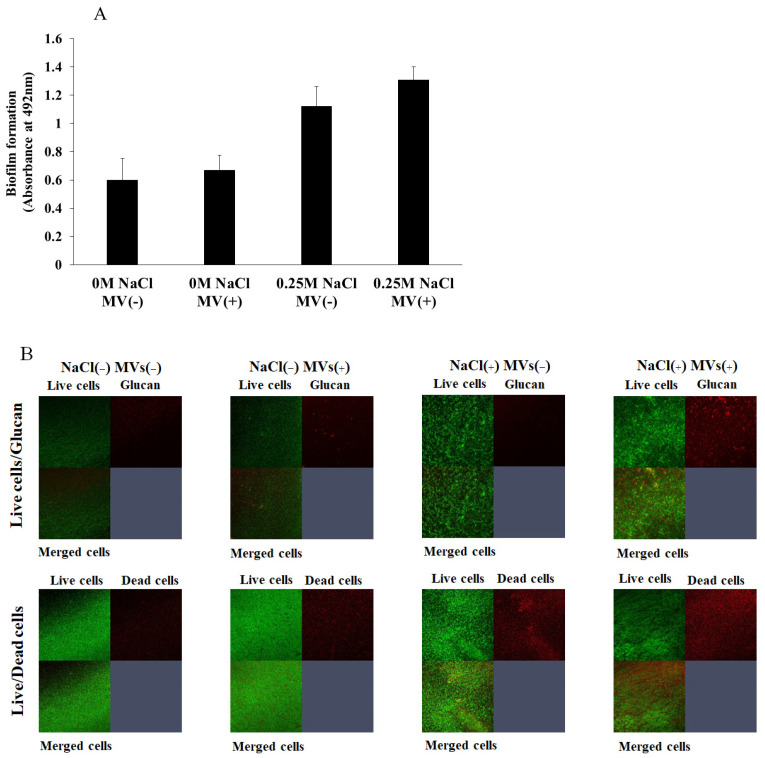
Effects of MVs on the formation of *S. aureus* biofilms with and without NaCl. *S. aureus* was inoculated in TSBs with and without 0.25 M NaCl to assess biofilm formation with and without *S. mutans* MVs (**A**). The data are presented as the means ± SDs of three independent experiments at 16 h after culture starts. An asterisk indicates that there is a significant difference between the two conditions in 0 M NaCl or 0.25 M NaCl in Student’ *t*-test (MVs vs. no MVs). The biofilms were treated by live/glucan and live/dead staining and observed by confocal microscopy (**B**). Upper row: upper left square indicates live cells biofilm. The upper right square indicates glucan or dead cells. The lower left square indicates biofilm merged with live cells and glucan or dead cells. Representative data from more than three independent experiments are presented in the images.

MVs are released upon Gram-positive bacteria cell lysis [38]. MVs are important for the development of *S. mutans* biofilms because they are associated with GTFs and induce soluble and insoluble glucan-dependent biofilm formation of other bacteria [23]. To determine whether MVs containing GTFs affect the formation of *S. aureus* biofilms, 0.25 M NaCl was added to *S. aureus* to assess biofilm formation in the presence of 0.25 mg/mL *S. mutans* MVs, as this concentration induced the greatest biofilm formation in TSBs (Figure 3B). *S. aureus* biofilm formation was significantly increased by the addition of 0.25 NaCl. Moreover, the addition of MVs slightly enhanced biofilm formation in 0.25 M NaCl, but the difference was not significant (Figure 5A). To observe glucan generation during *S. aureus* biofilm formation with MVs in TSB with 0.25 M NaCl, Alexa Fluor 647-dextran conjugates were added to the assay mixture, and the results were compared with those obtained without MVs. Glucan generation was clearly observed upon the addition of MVs in the presence of 0.25 M NaCl (Figure 5B). Therefore, the slight improvement in *S. aureus* biofilm formation with MVs might be dependent on the presence of glucan. Compared with the dead cells in the absence of 0.25 M NaCl, more dead cells were found in the biofilms in the presence of 0.25 M NaCl (Figure 5B). This may be because glucan can combine with dead cells to induce biofilm formation.

**Figure 6:**
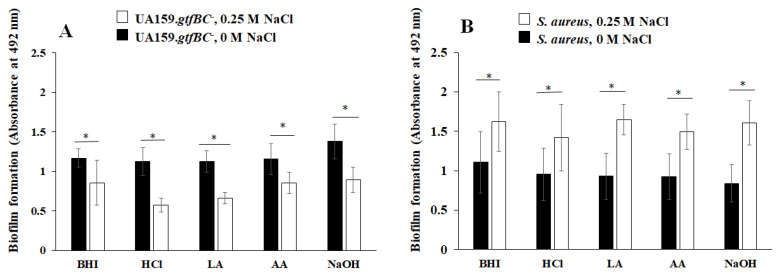
The effects of *S. mutans* MVs acquired after cultivation in media with various initial pH values. *S. mutans* UA159 *gtfBC*^−^ (**A**) and *S. aureus* (**B**) biofilms were cultivated in TSBs with and without MVs in media with an initial pH value of 6.0 prepared via the addition of HCl, lactic acid (LA), and acetic acid (AA), and in media with an initial pH value of 8.0 prepared via the addition of NaOH supplemented with 0 M NaCl and 0.25 M NaCl. The data are presented as the means ± SDs of three independent experiments at 16 h after culture starts. An asterisk indicates that there is a significant difference between the two conditions in the Student’ *t*-test (*: *p* < 0.05; NaCl vs. no NaCl).

To investigate different culture conditions, BHI media with initial pH values were prepared via the addition of various acids or NaOH, to which *S. mutans* UA159 was inoculated and cultivated overnight [34]. The pH values decreased in a time-dependent manner during culture. The MVs acquired from *S. mutans* cultures with an initial pH of 8.0 increased the protein volumes of GtfB and GtfC, which were comparable to the protein levels observed in the control (pH 7.2) [34]. The pH 6.0 media prepared with acetic acid produced MVs with greater protein volume of GtfC than those produced in pH 6.0 media prepared with HCl and lactic acid. The expression of *gtfB* and *gtfC* in *S. mutans* cultures was inhibited at pH 6.0 [36]. To observe the effects of different volumes of GtfB and GtfC in MVs, various MVs were applied during biofilm formation assays using *S. mutans* UA159.*gtfBC*^−^ and *S. aureus* with 0 and 0.25 M NaCl. The addition of NaCl inhibited the formation of *S. mutans* UA159.*gtfBC*^−^ biofilms in the presence of MVs (Figure 6A) but enhanced the formation of *S. aureus* biofilms in the presence of MVs (Figure 6B). The effect of NaCl on *S. aureus* biofilm formation was greater than the effects of different contents of Gtfs, as the volumes of GtfB and GtfC in the MVs did not affect *S. aureus* biofilm formation.

### 3.4. Effects of eDNA on Biofilm Formation

**Figure 7:**
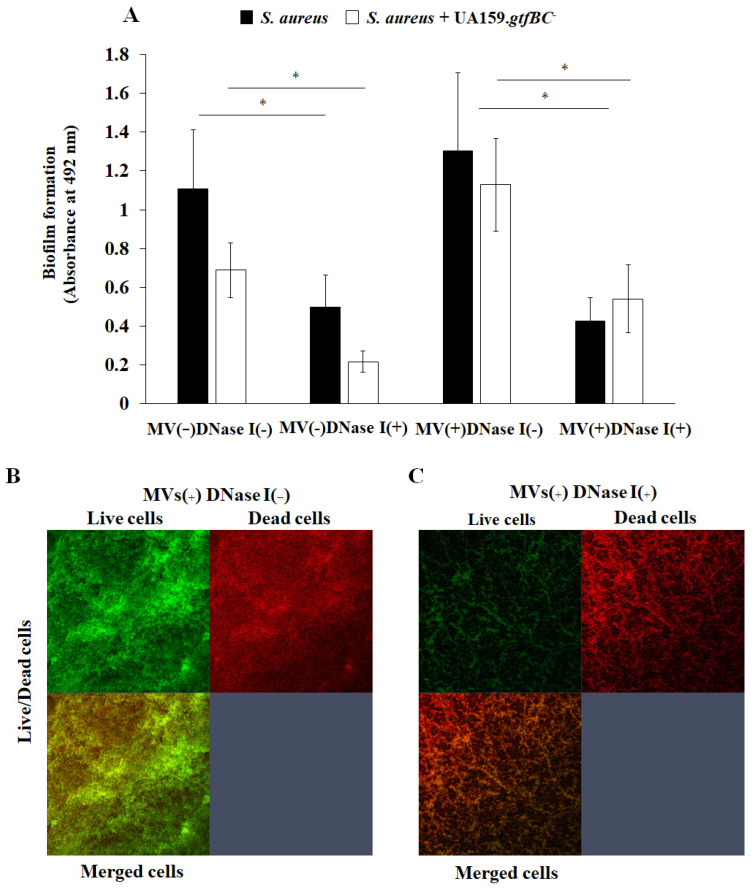
*S. aureus* or a mixture of *S. aureus* and *S. mutans gtfBC*^−^ was inoculated with and without MVs in TSBs with and without DNase I. The data represent the means ± SDs of three independent experiments at 16 h after start (**A**). An asterisk indicates that there are significant differences among the four conditions in the Student’ *t*-test (*: *p* < 0.05; DNase I vs. no DNase I). The MV-dependent *S. aureus* and *S. mutans gtfBC*^−^ mixed-species biofilms formed both with and without DNase I (**B**,**C**). The biofilms were treated through live/dead staining and observed via confocal microscopy. Upper row: upper left square indicates live cells in biofilm. The upper right square indicates dead cells. The lower left square indicates biofilm merged with live cells and dead cells. Representative data from more than three independent experiments are presented in the images.

To elucidate how eDNA affects biofilm formation, DNase I was added to assess the formation of *S. aureus* single-species biofilms and *S. aureus* and *S. mutans* UA159.*gtfBC*^−^ mixed-species biofilms with and without *S. mutans* MVs in the presence of 0.25 M NaCl (Figure 7). The formation of both the single- and mixed-species biofilms was significantly inhibited by DNase I. Notably, the formation of the mixed-species biofilms was enhanced by the addition of MVs, and the bacteria in the biofilm were significantly inhibited by DNase I (Figure 7A). To confirm glucan generation in the mixed-species biofilm, an Alexa Fluor 647-dextran conjugate was added during the biofilm formation assay along with MVs and 0.25 M NaCl. Glucan generation was detected, and the live cells in the biofilms were inhibited by the addition of DNase I, as observed via confocal microscopy (Figure 7B). Therefore, eDNA principally contributes to the formation of mixed-species biofilms in the presence of salt.

## 4. Discussion

The formation of both single- and mixed-species *S. aureus* biofilms, which are tolerant to high salt concentrations, increased with the addition of 0.25 M NaCl (Figure 3). When 0.25 M NaCl was added to TSBs containing 0.086 M NaCl, the final concentration of NaCl in the media was 0.336 M. The physiological salt concentration in the human body is 0.154 M NaCl [39], and this concentration of NaCl is used during fluid infusion. Increasing the NaCl concentration in food to the physiological concentration might stimulate eDNA-dependent *S. aureus* biofilm formation. Additionally, high salinity might increase the density and viscosity of bacterial cell suspensions [34], which may be a result of the ability of these cells to survive under high-salinity conditions. The high viscosity and density of hypersaline solutions result in suspended particles, such as MVs, having a reduced settling velocity [40]. The slower settling of the MVs may result in increased turbidity for *S. aureus* and increased efficiency of aggregation transport during mixed-species biofilm formation. Moreover, fructan, which is produced by *S. mutans* in the presence of sucrose, may affect biofilm formation because it enhances the viscosity of eDNA solutions and is necessary for *Bacillus subtilis* [41] and *S. mutans* biofilm formation [34]. It was thus hypothesized that the bacterial cells aggregate due to the increased density and viscosity of the cell suspension in the presence of eDNA and fructan under high-salinity conditions. When *S. mutans* is inoculated in a high-salinity environment with high osmotic pressure, the difference in osmotic pressure between the inside and the outside of the bacterial cell causes plasmolysis and death, which reduces the amount of biofilm that forms [42]. In contrast, *S. aureus*, which is salt-tolerant, increases its intercellular osmolality by storing glycine betaine intracellularly and incorporating osmolytes such as proline and taurine [43]. As a result, *S. aureus* survives by maintaining an equilibrium between the osmotic pressure inside and outside the cell and subsequently forms biofilms under the favorable high-salinity conditions mentioned above.

*S. mutans* produces three types of GTFs: GTF-I, encoded by *gtfB*; GTF-SI, encoded by *gtfC*; and GTF-S, encoded by *gtfD* [44,45]. In the presence of sucrose, the polysaccharide synthesized by GTF-I on the bacterial surface is composed of insoluble glucan rich in α1.3 linkages, and insoluble glucan is thought to be derived from bacterial aggregation [19,20]. Furthermore, GTF-SI, which is attached to the salivary pellicle-covered tooth surface, produces insoluble and soluble glucan rich in α1.3 and α1.6 linkages [21]. Aggregated bacteria, guided by insoluble glucan, attach to the tooth surface and fuse with glucan. *S. aureus* is an opportunistic pathogen that is associated with oral biofilms in the oral cavities of elderly people requiring care [7,8]. However, *S. aureus* does not express glucan-binding proteins (gbps), such as the gbps of *S. mutans* [21,46]. Soluble and insoluble glucans might not affect the adherence and aggregation of *S. aureus* because *S. aureus* differs from *S. mutans* in various ways. However, MVs containing GTFs slightly enhanced the formation of single-species *S. aureus* biofilms and mixed-species *S. aureus* and *S. mutans* UA159.*gtfBC*^−^ biofilms under high-salinity conditions. *S. mutans* MVs are associated mainly with GtfC [32], and *S. mutans* biofilms are enhanced by both the soluble and insoluble glucans in MVs. To investigate whether differences in the abundance of Gtfs in MVs affect *S. aureus* biofilm formation under high-salinity conditions, a biofilm formation assay with *S. aureus* and MVs with different amounts of Gtfs, as identified in a previous study [36], was performed. More Gtfs were loaded in MVs acquired in pH 8.0 media prepared via the addition of NaOH [36]. The pH of this medium did not decrease to less than 5.5 after 16 h of culture, and the expression of *gtfB* and *gtfC* was not inhibited by low pH. In pH 6.0 prepared via the addition of acetic acid, the protein volume of GtfC in the MVs was lower than that in pH 8.0 media but greater than that in pH 6.0 media prepared via the addition of HCl or lactic acid [36]. The pH decreases to below 5.0 after culture because lactic acid is produced, and the expression of *gtfB* and *gtfC* was inhibited by low pH. However, weak acids, such as acetic acid, can permeate bacterial membranes more readily than strong acids such as HCl and lactic acid. The addition of acetic acid induced a greater increase in the GtfC volume than the addition of HCl and lactic acid did, resulting in oral biofilms with different activities [36]. However, different volumes of GtfB and GtfC in the MVs did not affect *S. aureus* biofilm formation (Figure 6). Therefore, the presence of both soluble and insoluble glucans might support the physical adherence and aggregation of *S. aureus* stimulated by excess salt (0.25 M NaCl), whereas the difference in the volumes of soluble and insoluble glucans did not affect the volume of the biofilm formed.

Mature *S. aureus* biofilms are sensitive to the external addition of DNase I, indicating that eDNA is a structural component of the *S. aureus* biofilm matrix [47]. Owing to the negative charge of DNA, eDNA may participate in the early adhesion and maturation stages of biofilm formation by participating in electrostatic interactions and playing a basic role in the structural integrity of the biofilms [48]. In this study, biofilm formation by live bacterial cells was induced mainly by the presence of eDNA and was not influenced by glucan under high-salinity conditions in TSBg. The presence of NaCl preferentially induces eDNA-dependent live *S. aureus* biofilm formation via different mechanisms than those of glucan generation by *S. mutans* MVs. The opportunities and mechanisms by which *S. aureus* produces DNA are unknown, but DNA synthesis is required when a single *S. aureus* cell initially attaches to the surface in the absence of glucan.

Cyclic di-adenosine monophosphate (c-di-AMP) is a recently discovered secondary messenger that is produced predominantly by Gram-positive bacteria [49,50,51,52]. c-di-AMP production is critical for the growth of *S. aureus* in chemically defined media and in rich media supplemented with additional sodium or potassium chloride [53]. The influence of c-di-AMP on biofilms has been shown and may play an important role in the persistence of *S. aureus* biofilms under high-salinity conditions [54]. The production of c-di-AMP promotes K^+^ and cell wall homeostasis, biofilm formation, and virulence but sensitizes the cells to osmotic stress [55,56,57]. Elevated c-di-AMP concentrations may promote *S. aureus* biofilm formation under high-salinity conditions, as previously shown in several streptococci [58,59].

Compared with single-species *S. aureus* biofilms, whole-cell *S. mutans* biofilms reduced the formation of mixed-species biofilms (Figure 7A). In contrast, *S. mutans* MVs slightly enhanced dead cell-dependent biofilm formation (Figure 5B). Glucans in MVs might enhance the associations between live and dead *S. aureus* cells under high-salinity conditions. In contrast, in the presence of glucose, *S. aureus* may be able to attach to and colonize old biofilms containing dead bacteria because dead cells do not produce antibacterial substances such as acids or bacteriocin [7]. When the commensal bacteria in the oral cavity are alive and colonizing, opportunistic pathogens such as *S. aureus* are less likely to colonize [60]. This is also why young people with sufficient normal oral flora have a low incidence of *S. aureus* infection.

Thus, MVs, eDNA, and dead cells from *S. mutans* enhance *S. aureus* biofilm formation in the presence of high concentrations of salt. The sugar (sucrose and glucose) ingestion-induced *S. mutans* biofilm may be a risk factor for dental caries with *S. aureus* in the elderly and bedridden, who typically have high physiological concentrations of NaCl. Therefore, to avoid dental caries via infection by opportunistic pathogens, such as *S. aureus*, and to maintain oral and systemic health, sucrose, glucose, and NaCl intake should be limited.

## 5. Conclusions

The presence of *S. mutans* might be a risk factor for *S. aureus* infection in the oral cavity because the formation of single-species *S. aureus* biofilms and *S. aureus* mixed-species biofilms is enhanced by MVs, extracellular DNA, and dead *S. mutans* cells in tryptic soy broth with sucrose, glucose, and high concentrations of salt, including NaCl. The glucosyltransferases in *S. mutans* MVs slightly enhanced the association between *S. aureus* and dead cells under high-salinity conditions in TSBs. Biofilm formation by live bacterial cells was induced mainly by the presence of extracellular DNA. Therefore, to avoid dental caries via infection by opportunistic pathogens, such as *S. aureus*, and to maintain oral and systemic health, sucrose, glucose, and NaCl intake should be limited.

## Data Availability

The original contributions presented in this study are included in the article. Further inquiries can be directed to the corresponding author.
